# Constitutive heterochromatin controls nuclear mechanics, morphology, and integrity through H3K9me3 mediated chromocenter compaction

**DOI:** 10.1080/19491034.2025.2486816

**Published:** 2025-04-09

**Authors:** Gianna Manning, Andy Li, Nebiyat Eskndir, Marilena Currey, Andrew D. Stephens

**Affiliations:** a Biology Department, University of Massachusetts Amherst, Amherst, MA, USA; b Molecular and Cellular Biology, University of Massachusetts Amherst, Amherst, MA, USA

**Keywords:** Chromocenter, heterochromatin, nuclear blebbing, nuclear mechanics, nuclear structure

## Abstract

Aberrant nuclear morphology is a hallmark of human disease and causes nuclear dysfunction. Perturbed nuclear mechanics via reduced heterochromatin weakens the nucleus resulting in nuclear blebbing and rupture. While the role of heterochromatin is known, the separate roles of constitutive heterochromatin methylation states remains elusive. Using MEF and HT1080 cells, we isolated the individual contribution of constitutive heterochromatin H3K9 methylation states through histone methyltransferase inhibitors. Inhibition of SUV39H1 via Chaetocin downregulates H3K9 trimethylation (me3), while inhibition of G9a via BIX01294 downregulates H3K9 dimethylation (me2). Overall, the loss of H3K9me3 increased nuclear blebbing and rupture in interphase nuclei due to decreased nuclear rigidity from decompaction of chromocenters. Oppositely, loss of H3K9me2 decreased nuclear blebbing and rupture with increased nuclear rigidity and more compact chromocenters. We show that facultative heterochromatin and HP1α are non-essential for chromocenter compaction. Constitutive heterochromatin provides essential nuclear mechanical support to maintain nuclear shape and integrity through chromocenter compaction.

## Introduction

The nucleus is the organelle that houses the genome and its essential functions. Chromatin and lamins are the two major mechanical components of the nucleus. These mechanical elements maintain nuclear shape and integrity by resisting antagonistic actin contraction and confinement [1–7] and microtubule dynamics [8,9]. Weakening of the nucleus through loss of chromatin compaction or lamins leads to abnormal nuclear morphology [10]. A type of abnormal nuclear morphology is a herniation of the nucleus called a nuclear bleb, hallmarked by a one-third decrease in DNA density [11,12]. The increased curvature caused by a nuclear bleb leads to >95% of them resulting in nuclear rupture [13–15]. Loss of nuclear integrity is well documented to cause increased DNA damage [7,15–21], changes in transcription [22–24], and loss of cell cycle control [25], and death [19,26]. Overall nuclear mechanics controls nuclear shape and integrity to maintain function.

Chromatin is a key mechanical element that specifically provides physical resistance via chromatin compaction largely through heterochromatin. Heterochromatin is maintained through histone modification states which can largely be separated into constitutive and facultative. Constitutive heterochromatin is consistent heterochromatin in all cell types representing structures like telomeres and centromeres, while facultative heterochromatin facilitates variable silencing of underlying genes dependent on cell type and differentiation state [27]. While there are many histone modifications, methylation at two major modification sites H3K9 (constitutive) and H3K27 (facultative) have been shown to be important to both nuclear mechanics and morphology broadly [14,21,28–31]. However, constitutive heterochromatin consists of H3K9 dimethylation (H3K9me2) and trimethylation (H3K9me3) that have distinct roles in chromatin and cell biology [32], which could lead to different roles in nuclear mechanics. The separate roles of these two histone methylation modifications on nuclear morphology, shape, and mechanics remain unknown.

Recent work has revealed two nuclear mechanics subcomponents including the linkages within chromatin such as HP1α [30,31] and chromatin conformation connections [33] and between chromatin and the nuclear periphery [34]. H3K9me3 is enriched in internally located chromocenters and the nuclear periphery [35,36]. H3K9me3 is also the preferred binding site for HP1α [37,38] with that preference being about 3-fold stronger than H3K9me2. HP1α acts as a chromatin linker through its dimerization and binding of two different nucleosomes via H3K9 methylation binding [39,40] that provides mechanical resistance and nuclear morphological support [30,31]. However, HP1α and constitutive heterochromatin have been reported to have separable contributions to nuclear mechanics [30]. Thus, it will be important to continue to separate the role of constitutive heterochromatin from HP1α in nuclear mechanics, morphology, and integrity at these interaction sites of the chromocenters and nuclear periphery.

To determine the separate contributions of constitutive heterochromatin H3K9 di- and tri-methylation we utilized specific inhibitors of histone methyltransferases G9a and SUV39H1, respectively. Using immunofluorescence, we demonstrated that these inhibitors independently reduce levels of H3K9me2 and H3K9me3 and compared this to the established facultative heterochromatin histone methyltransferase inhibitor, DZNep to decrease H3K27me3. Next, we assayed nuclear shape and integrity dynamics due to the loss of either H3K9me2 or H3K9me3 using NLS-GFP. The accumulation of NLS-GFP in the nucleus provides a fluorescence readout of both nuclear shape when compartmentalized and nuclear rupture when NLS-GFP diffuses into the cytoplasm. Nuclear shape and integrity are dependent on nuclear mechanics, and thus we performed dual micropipette micromanipulation nucleus force-extension measurements that provide separate measures of chromatin and lamin contribution to nuclear mechanics. Finally, we measured chromocenter compaction and nuclear periphery enrichment of chromatin to elucidate which structural features were affected by the loss of either H3K9me2 or H3K9me3. Overall, we report that H3K9me3 is a major determinant of constitutive heterochromatin through maintaining chromocenter compaction to provide mechanical support necessary to maintain both nuclear morphology and integrity.

## Results

### Inhibition of different methyltransferases provide specific modulation of H3K9me2 and H3K9me3

**Figure 1.**
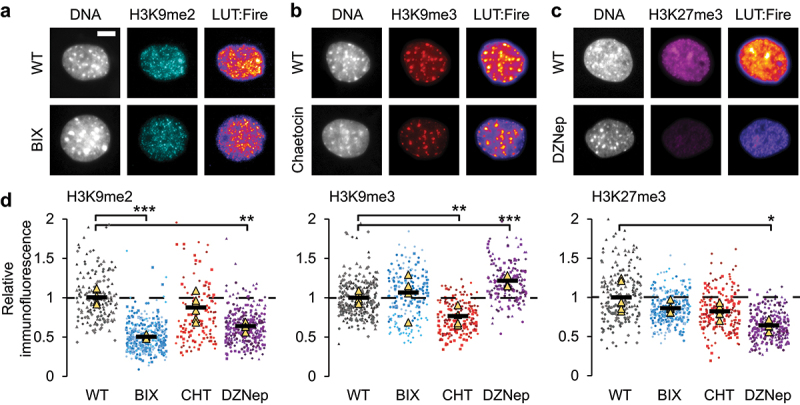
Heterochromatin histone modification states can be modulated distinctly. (a-c) Example images of MEF nuclei DNA stained by Hoechst (gray) and immunofluorescence for (a) H3K9me2 (blue), (b) H3K9me3 (red), and (c) H3K27me3 (purple). For each immunofluorescence marker there is an example image of wild type (WT) and inhibitor including BIX01294, Chaetocin (CHT), or DZNep treatment for 16 h. (d) Graphs of immunofluorescence relative to wild type for all inhibitors for H3K9me2, H3K9me3, and H3K27me3. Each condition consists of six replicates n averages > 20 nuclei each. One-way ANOVA multiple comparison between wild type and each treatment with *p* values reported as *< 0.05, **< 0.01, ***< 0.001, or ns denotes no significance, *p* > 0.05. Error bars represent standard error. Scale bar = 10 µm.

To determine the different contributions of histone modifications associated with constitutive heterochromatin, we inhibited different methyltransferases. We used immunofluorescence to measure changes in histone modifications after treatment of mouse embryonic fibroblasts (MEFs) with different methyltransferase inhibitors. We specifically measured the levels of constitutive heterochromatin histone modifications H3K9 dimethylation (H3K9me2) and trimethylation (H3K9me3), and facultative heterochromatin marker H3K27 trimethylation (H3K27me3). Upon treatment of MEF cells with BIX01294, an inhibitor of the G9a methyltransferase [41], we measured only a significant decrease in H3K9me2 (Figure 1(a,d)), in agreement with previous published work [42]. Treatment of MEF cells with Chaetocin, an SUV39-H1 methyltransferase inhibitor [43], resulted in decreased H3K9me3 (Figure 1(b,d)) in agreement with previously published reports [44–46]. Chaetocin did not significantly alter H3K9me2 or H3K27me3 levels (Figure 1(d)). Finally, MEF cells treated with DZNep, a broad methyltransferase inhibitor, resulted in loss of H3K27me3 and H3K9me2 while also showing an increase in H3K9me3 (Figure 1(c,d)), in agreement with primary literature of this inhibitor [47]. Our immunofluorescence data revealed no changes in active phospho-myosin light chain 2 (pMLC2) levels that cause actin contraction and slight but insignificant changes in global lamin A/C levels and HP1α (Supplemental Figure S1a-c). Taken together, we show that we can distinctly decrease the levels of different key histone modifications via the use of different methyltransferase inhibitors.

### Loss of H3K9me3 results in increased nuclear blebbing and rupture while loss of H3K9me2 does not

**Figure 2.**
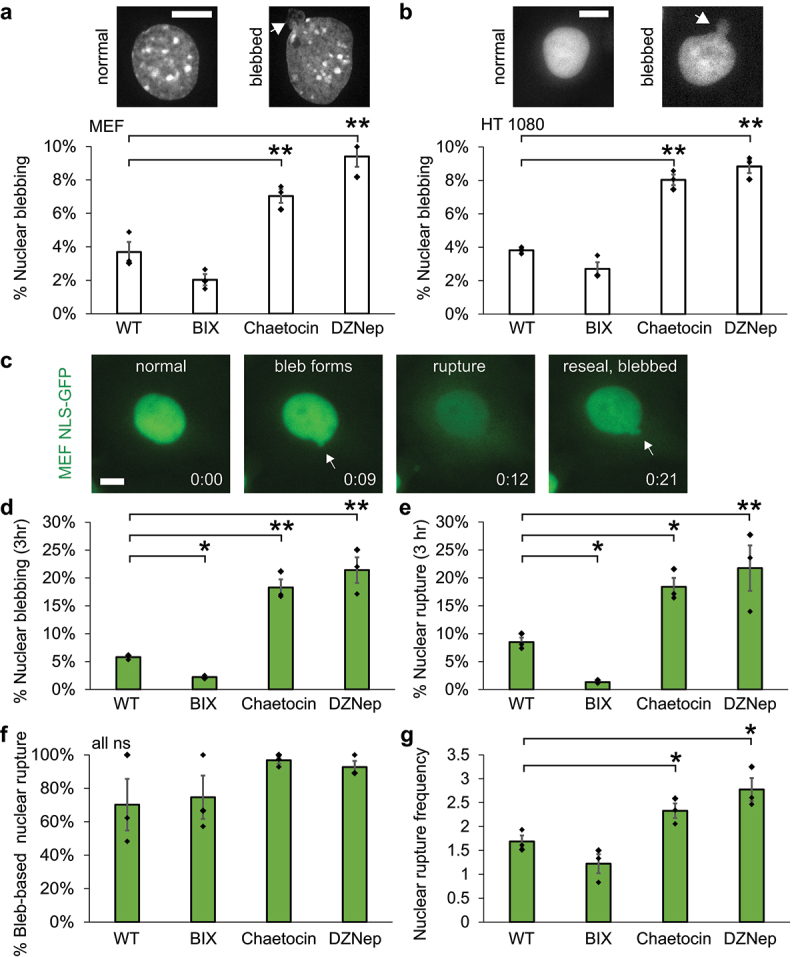
Nuclear blebbing and rupture increase upon loss of H3K9me3 while loss of H3K9me2 decreases this effect. (a) Example images of a normal shaped and blebbed MEF nucleus DNA stained by Hoechst. Graph of percentage of blebbed nuclei from population imaging after 16 h of treatment. Each condition consists of three biological replicates n > 60 nuclei each. (b) Example images of a normal shaped and blebbed HT1080 nucleus via H2B-mcherry. Graph of percentage of blebbed nuclei from live cell imaging after 16 h of treatment. Each condition consists of three technical replicates n > 40 nuclei each. (c) Example images of nuclear bleb formation and rupture upon timelapse imaging of MEF NLS-GFP cells over 3 h. Time between images indicated by time stamp hour:minute. Graphs of 3 h timelapse imaging (d) percent nuclear blebbing, (e) percent nuclear rupture, (f) percent bleb-based nuclear rupture, and (g) nuclear rupture frequency for conditions wild type (WT), BIX01294 (BIX), Chaetocin, and DZNep treatment over 16 h. Each condition consists of three biological replicates n > 180 nuclei each replicate and > 600 nuclei total for nuclear blebbing and rupture. Two-tailed unpaired Student’s t-test between wild type and each treatment with *p* values reported as *< 0.05, **< 0.01, ***< 0.001, or ns denotes no significance, p > 0.05. Two-tailed unpaired Student’s t-test between Chaetocin and DZNep were all p > 0.05. Error bars represent standard error. Scale bar = 10 µm.

Previous work has revealed histone modifications states determine nuclear morphology [14,21,48,49]. To determine the relevant histone modifications that provide support for nuclear shape we compared loss of H3K9 di- and tri-methylation. First, we measured population levels of nuclear blebbing after 16-h inhibitor treatments. Wild type MEFs present a low rate of nuclear blebbing at 4% (Figure 2(a)). Treatment of MEF cells with BIX01249 to decrease H3K9me2 resulted in an insignificant nuclear blebbing decrease to 2% (*p* > 0.05, Figure 2(a)). Oppositely, Chaetocin-treated MEF cells which significantly decreased H3K9me3 resulted in a significant increase in nuclear blebbing to 7%. DZNep-treated cells to decrease H327me3 also significantly increased nuclear blebbing to 9% (Figure 2(a)), in agreement with previous published studies [14,21]. We then treated a different cell line to determine if this effect was broad. HT1080 human fibrosarcoma cells show a base 4% nuclear blebbing imaged by H2B-mcherry which does not change with BIX01294 treatment but did significantly increase upon treatment with Chaetocin or DZNep (Figure 2(b)). Thus, both MEF and HT1080 cell lines show similar changes in nuclear blebbing upon different histone modification alterations. Finally, lack of changes in pMLC2 immunofluorescence indicated that actin contraction, a known regulator of nuclear blebbing [5,7], is not responsible for these changes (Supplemental Figure S1a). Overall, loss of constitutive heterochromatin H3K9me2 leads to no change, while the loss of constitutive heterochromatin marker H3K9me3 or facultative heterochromatin marker H3K27me3 causes increased nuclear blebbing.

Nuclear blebbing is important because it drives nuclear rupture [10,50]. To determine if nuclear blebs generated by Chaetocin-induced H3K9me3 loss results in nuclear rupture, we timelapse imaged nuclear blebbing and rupture in MEF NLS-GFP cells over 3 h with 3-min intervals after 16 h of inhibitor treatment (Figure 2(c)). Wild type MEF NLS-GFP cells presented a low rate of 6% nuclear blebbing and 8% nuclear rupture over this 3-h timelapse interval (Figure 2(d,e)). In agreement with decreased static nuclear blebbing, timelapse imaging of BIX01249-treated nuclei to decrease H3K9me2 revealed a significant decrease in nuclear blebbing to 2% and ruptures to 1%. Timelapse imaging revealed that the loss of H3K9me3 via Chaetocin resulted in increased nuclear blebbing and rupture to 18%. This increase was similar to the loss of facultative heterochromatin H3K27me3 via DZNep (*p* > 0.05, Figure 2(d,e)). To determine the source of nuclear rupture, we then measured the percentage of nuclear ruptures that were specifically due to nuclear blebbing. Across all conditions, the majority of nuclear ruptures stem from nuclear blebs (Figure 2(f)). Finally, we measured the nuclear rupture frequency by tracking nuclei that rupture for how many times those nuclei rupture. Both wild type and BIX nuclei presented a similar 1.7 and 1.5 nuclear rupture frequency while loss of H3K9me3 via Chaetocin causes a significant increase to 2.5, which is similar to DZNep-treated nuclei (Figure 2(g)). Thus, the loss of H3K9me2 confers stable nuclear shape and integrity, while the loss of H3K9me3 significantly increased in nuclear blebbing and rupture similar to the loss of H3K27me3 facultative heterochromatin.

### Nuclear blebbing upon loss of H3K9me3 is an interphase behavior separate from its effect on mitotic division

**Figure 3.**
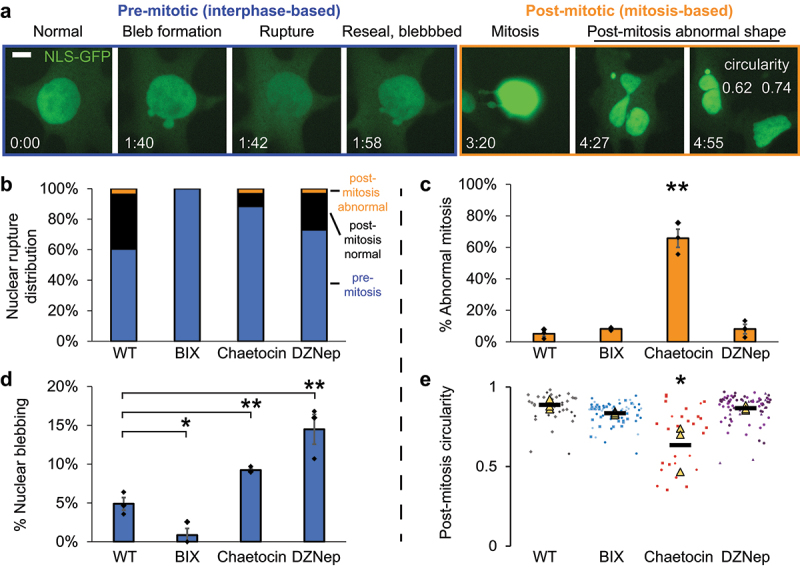
Nuclear blebbing and rupture upon loss of H3K9me3 is due to interphase-based behaviors and not dependent on mitotic failures. (a) Example images of MEF NLS-GFP cells treated with Chaetocin and imaged for 16 h. Pre-mitosis the normal shaped nucleus forms nuclear blebs and ruptures before going through an abnormal mitosis resulting in abnormally shaped daughter nuclei post-mitosis which can be measured by decreased circularity. Time between images indicated by time stamp hour:minute. (b) Nuclear rupture distribution categorized for occurring pre-mitosis, post normal mitosis, post abnormal mitosis. Nuclear ruptures of three technical replicates WT (n = 13, 6, 8) BIX01294 (n = 1, 1, 0) Chaetocin (n = 10, 4, 11), DZNep (n = 16, 18, 27). (c) Graph of the percentage of abnormal mitosis for wild type (WT), BIX01294 (BIX), Chaetocin, and DZNep treatment over the later 16 h of a 24-h treatment. Each condition consists of three biological replicates n averages > 30 mitotic events. (d) Graph of the percentage of interphase-based nuclear blebbing for (WT), BIX01294 (BIX), Chaetocin, and DZNep treatment over 16 h. Each condition consists of three biological replicates with n > 50 nuclei each. (e) Post-mitosis nuclear circularity measured 15 min after NLS-GFP nucleus accumulation. Each condition consists of three technical replicates n > 25 nuclei total. Two-tailed unpaired Student’s t-test between all conditions with *p* values reported as *< 0.05, **< 0.01, ***< 0.001, or ns denotes no significance, p > 0.05. For panels C and E Chaetocin is statistically significantly different from all other conditions. Error bars represent standard error. Scale bar = 10 µm.

Loss of constitutive heterochromatin marker H3K9me3 is well reported to cause mitotic failure [51–53]. It is possible that nuclear blebbing could be due to mitotic failures in Chaetocin-treated cells. To separate the role of H3K9me3 in interphase-based nuclear morphology and its role in mitosis, we conducted long-term timelapse imaging. Cells were incubated in the inhibitor for 8 h prior to imaging. Live cells were then imaged over 16 h with continued treatment, allowing us to measure if nuclear blebbing and rupture occurs independent of mitosis (pre-mitotic) or because of mitosis (post-mitotic, Figure 3(a)). Consistently, we find that nuclear ruptures occur mostly in the interphase nucleus before reaching its first mitosis across all conditions (Figure 3(b)). Furthermore, we tracked normal mitosis and abnormal mitosis. Nuclear rupture was rarely (<5%) caused by abnormal mitosis. This is especially important in Chaetocin-treated cells where loss of H3K9me3 caused mitotic failure in 60% of the mitoses imaged (Figure 3(c)). Loss of H3K9me2 and H3K27me3 did not disrupt normal mitosis relative to wild type. We further confirmed that over the 16-h nuclear blebbing percentages closely resembled 3-h timelapse data (Figure 3(d) vs Figure 2(d)). Interestingly, abnormal mitosis resulted in abnormally shaped daughter nuclei measured by a significant decrease in nuclear circularity (Figure 3(e)), in agreement with previous reports [54]. Thus, the loss of H3K9me3 causes interphase-based nuclear bleb formation and rupture pre-mitosis, while abnormal mitosis resulted in abnormal (less circular) daughter nuclei.

### H3K9me3 is an essential component of nuclear mechanics

**Figure 4.**
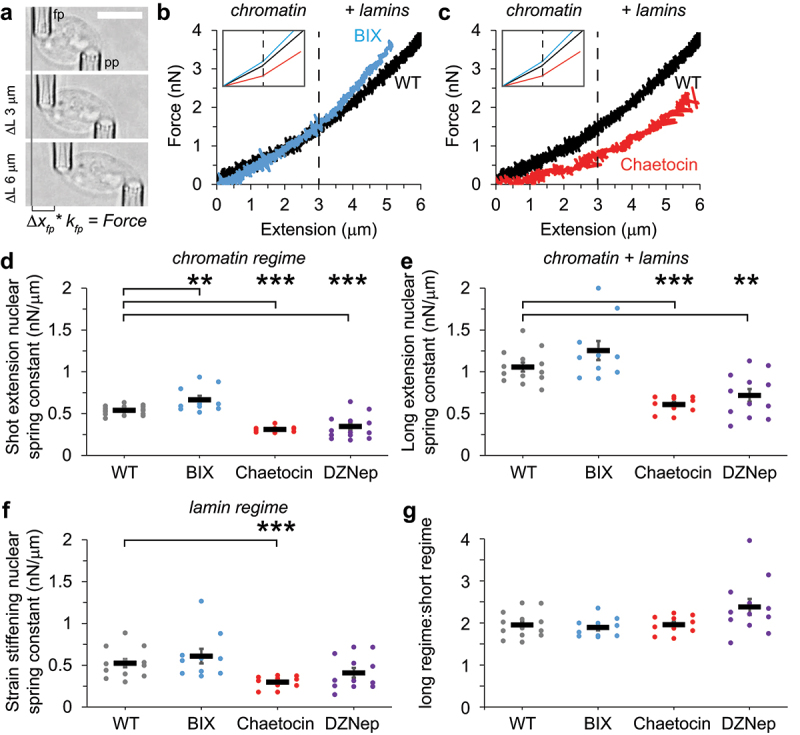
Nuclear stiffness increases upon loss of H3K9me2 and decreases upon loss of H3K9me3. (a) Example images of a single isolated nucleus dual micropipette micromanipulation force-extension measurement experiment. These nuclei are isolated from MEF vimentin null nuclei to aid clean isolation from live cells. The pull pipette (pp) extends the nucleus as the force pipette’s (fp) deflection multiplied by its premeasured bending constant measures force. (b, c) Example nucleus force–extension curves for (b) BIX01294 (blue, decreased H3K9me2) and (c) Chaetocin (red, decreased H3K9me3) relative to an example wild type (WT, black). Dotted line at 3 µm denotes separation of short and long extension regimes, previously shown to reflect respectively the chromatin and chromatin + lamin mechanical contributions [29,55]. Inset shows the relative slopes with provide the nuclear spring constant, k = f/δl. (d-f) Average nuclear spring constants for (d) short extension <3 µm, (e) long extension >3 µm, and (f) strain stiffening via long minus short extension regimes for wild type (WT, gray, n = 18 short and 13 long), BIX01294 (BIX, blue, n = 10), Chaetocin (red, n = 10), and DZNep (purple, n = 13 short and 12 long). (g) Graph of the ratio of the long extension and short extension nuclear spring constant for wild type (WT), BIX01294 (BIX), Chaetocin, and DZNep. Mann-Whitney U test between wild type and each treatment were run for panels d-f and a one-way ANOVA multiple comparison was run for panel G with *p* values reported as *< 0.05, **< 0.01, ***< 0.001, or ns denotes no significance, p > 0.05. Chaetocin and DZNep were statistically similar for all graphs d-g, p > 0.05. Error bars represent standard error. Scale bar = 10 µm.

Histone modifications have an essential role in determining nuclear mechanics through their roles in chromatin. Dual micropipette micromanipulation force extension measurements provide the unique ability to separate the mechanical contributions of chromatin and lamins [29,55]. Using micropipettes, we can isolate a single nucleus from a living cell and suspend it between two micropipettes. To aid isolation, we use a MEF vimentin null nucleus which has been consistently shown to have similar nuclear blebbing and rupture behavior as wild type [14,21,22]. The pull micropipette pulls the nucleus to cause extension while the force micropipette’s deflection multiplied by its pre-measured bending constant allows us to calculate force (Figure 4(a)). The force (nN) vs. extension (µm) trace provides a nuclear spring constant (nN/µm). Furthermore, this trace can be divided into the short extension chromatin-dominated regime (<3 µm) and a long extension regime (>3 µm) in which the nucleus strain stiffens due to lamin A (Figure 4(b,c) [29,55]). These different regimes have been verified by other force measurements techniques such as single-plan illumination microscopy combined with atomic force microscopy and optical tweezers [56,57].

Isolated MEF nuclei measured by dual micropipette micromanipulation have a short extension chromatin-based nuclear spring constant of 0.54 ± 0.01 nN/µm and a long extension regime represented by both chromatin and lamin contributions at 1.08 ± 0.05 nN/µm. Upon decreased H3K9me2 via BIX01249 treatment the short extension nuclear spring constant significantly increased to 0.66 ± 0.05 nN/µm, while the both the long extension and strain stiffening nuclear spring constant did not significantly change (Figure 4(b,d-f)). This increased chromatin-based rigidity upon H3K9me2 loss agrees with the data above reporting less nuclear blebbing and rupture (Figure 2(d,e)). Oppositely, loss of H3K9me3 via Chaetocin treatment resulted in a significant decrease in the short extension nuclear spring constant to 0.31 ± 0.01 nN/µm, suggesting it is disrupting chromatin-based nuclear mechanics (Figure 4(c,d)). Loss of H3K9me3 also resulted in a significant decrease in the long extension nuclear spring constant to 0.61 ± 0.03 nN/µm that measures joint chromatin and lamin contribution (Figure 4(e)). One possibility is that H3K9me3’s major contribution to chromatin-based nuclear mechanics is sufficient to also decrease this combined regime, though alternatively lamin-based nuclear mechanics might also be impacted. To determine if H3K9me3 loss also affects lamin-based nuclear mechanics, we measured the strain stiffening nuclear spring constant by subtracting the short extension nuclear spring constant from the long extension nuclear spring constant. Loss of H3K9me3 resulted in a significant decrease in the strain stiffening nuclear spring constant that has been previously attributed to lamins (WT 0.52 ± 0.05 nN/µm vs. Chaetocin 0.30 ± 0.02 nN/µm, Figure 4(f)). This is an interesting finding given that lamin A/C immunofluorescence does not decrease in Chaetocin treated cells (Supplemental Figure S1b). H3K9me3 contributes to both the chromatin (short) and lamin (long) regimes of nuclear mechanics.

It has been previously established that loss of facultative heterochromatin via DZNep treatment results in decreased nuclear mechanics through its role in supporting chromatin-based short extension nuclear spring constant [14,21]. We aimed to determine if the changes in short, long, and strain stiffening nuclear spring constants were similar upon loss of facultative heterochromatin. Upon the loss of H3K27me3 with DZNep treatment the short extension nuclear spring constant decreased similar to Chaetocin dropping to 0.32 ± 0.04 nN/µm (Figure 4(d)). Measurement of the long extension nuclear spring constant also revealed a significant drop from wild type to 0.72 ± 0.08 (Figure 4(e)). Different from the loss of constitutive heterochromatin H3K9me3, loss of H3K27me3 did not significantly change the strain stiffening regime from wild type (WT 0.52 ± 0.05 nN/µm vs. DZNep 0.41 ± 0.06 nN/µm, *p* > 0.05, Figure 4(f)). In agreeement, measurements of lamin A/C levels via immunofluorescence reveal that DZNep treated nuclei have no significant change in lamin A/C levels (Supplemental Figure S1b). One possibility is that chromatin and lamin strain stiffening is a maintained ratio. Measuring long divided by short regime nuclear spring constant reveals that long:short (chromatin + lamin:chromatin) ratio remains similar across all conditions suggesting that lamin-based strain stiffening could be partially reliant on the base mechanical resistance of chromatin (Figure 4(g)). Taken together, the loss of constitutive heterochromatin H3K9me3 affects both chromatin (short) and plus lamin (long) regimes, while H3K9me2 and H3K27me3 only modulate chromatin-based nuclear mechanics.

### Loss of H3K9me3 causes decompaction of chromocenters but no change in peripheral localization

Our measurements of nuclear spring constants lead to a hypothesis that depletion of H3K9me2 and H3K9me3 have opposite impacts on the constitutive heterochromatin structure. First, we measured constitutive heterochromatin at chromocenters, internal nuclear puncta. To understand the mechanism underlying decreased nuclear mechanics upon loss of H3K9me3, we imaged then measured chromocenter size and intensity to generate an effective compaction of the chromocenters.

**Figure 5.**
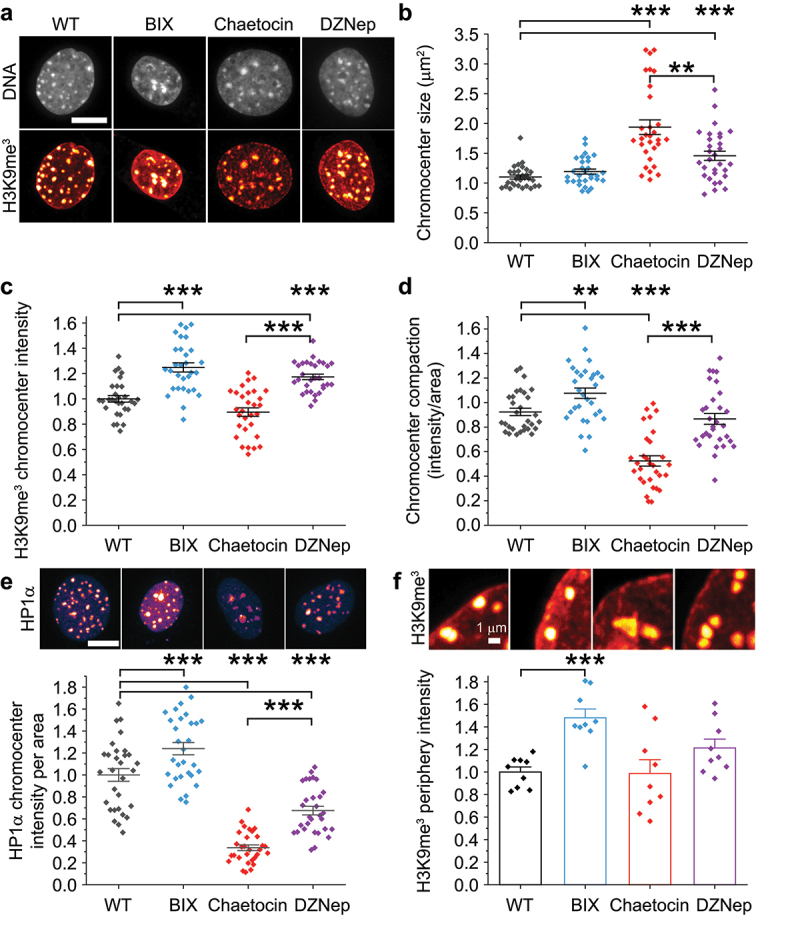
Chromocenter compaction increases upon loss of H3K9me2, decreases upon loss of H3K9me3, and is unchanged by loss of H3K27me3. (a) Example images of MEF nuclei DNA stained by Hoechst (gray) and immunofluorescence for H3K9me3 (red) for wild type (WT, gray), BIX01294 (BIX, blue), Chaetocin (red), and DZNep (purple). Scale bar = 10 µm. (b, c, d) Graphs of (b) chromocenter size and (c) chromocenter average H3K9me3 intensity used to calculate (d) chromocenter compaction for wild type (WT), BIX01294 (BIX), Chaetocin, and DZNep. The raw data for each chromocenter measured is graphed as a heatmap in Supplemental Figure S2. (e) Example images and graph of relative HP1α chromocenter intensity per area and (f relative H3K9me3 peripheral intensity for wild type (WT), BIX01294 (BIX), Chaetocin, and DZNep. Scale bar = 10 µm for panel E images and 1 µm for panel F images. Each condition consists of 30 nuclei each measuring n > 10 chromocenters. Mann-Whitney U test for panels B-D and one-way ANOVA multiple comparison for panels e-f between wild type and each treatment and Chaetocin compared to DZNep with *p* values reported as * < 0.05, ** < 0.01, *** < 0.001, or no asterisk denotes no significance, p > 0.05. Error bars represent standard error.

In support of our hypothesis, the loss of H3K9me2 had opposite effects on chromocenter compaction compared to H3K9me3. Upon decreased H3K9me2 via BIX01294 we measured no change in chromocenter size but a significant increase in intensity of H3K9me3 staining. This provided an overall increase in chromocenter compaction calculated by intensity per area, suggesting that loss of H3K9me2 resulted in over-compaction of this constitutive heterochromatin structure. Oppositely, loss of H3K9me3 via Chaetocin increased chromocenter size (Figure 5(a,b)), evidenced by sprawling chromocenters. This increase in size led to decreased chromocenter intensity per area, resulting in a 50% decrease in the effective chromocenter compaction relative to wild type (Figure 5(c,d); Supplemental Figure S2). DNA chromocenter compaction also showed a significant increase for BIX and decrease for Chaetocin (Supplemental Figure S2e). Interestingly, loss of H3K9me3 did not significantly change nuclear size compared to wild type but did decrease the overall number of chromocenters, further highlighting its importance in chromocenter structure (Supplemental Figure S2f,g). Overall, H3K9me2 loss increased chromocenter compaction, while the loss of H3K9me3 decreased chromocenter compaction.

Decreased H3K27me3 facultative heterochromatin via DZNep treatment provides a unique view into these structures. DZNep-treatment resulted in both an increased chromocenter size and intensity. This proportional increase in both parameters means that the effective compaction of chromocenters was not significantly altered by decreasing facultative heterochromatin (Figure 5). DNA chromocenter compaction also did not significantly change in DZNep-treated cells (Supplemental Figure S2e). Thus, the constitutive heterochromatin H3K9me2 and H3K9me3 levels modulate chromocenter compaction, while facultative heterochromatin is dispensable.

H3K9me3 is bound by a known mechanical component HP1α. To determine if changes in chromocenter compaction could be due to loss of HP1α, we measured its intensity per area (Figure 5(e)). HP1α levels did not change significantly throughout the nucleus (Supplemental Figure S1c) and thus measurements of HP1α at the chromocenter reflect local enrichment. HP1α chromocenter intensity per area increased significantly upon BIX-based loss of H3K9me2 but decreased significantly in both Chaetocin loss of H3K9me3 and DZNep loss of H3K27me3. Taken together, the lack of change in chromocenter compaction in DZNep with loss of HP1α suggests that the loss of HP1α alone is insufficient to alter chromocenter compaction or size. Dual treatment of BIX+DZNep returned nuclear blebbing as well as H3K9me3 and HP1α intensity per area to wild type levels (Supplemental Figure S3), suggesting that increased chromocenter compaction via BIX can rescue loss of facultative heterochromatin via DZNep. This data suggests that H3K9me2 is an antagonist and H3K9me3 is essential contributor to chromocenter compaction, while HP1α is not essential in agreement with other reports [58].

A non-mutually exclusive possibility is that changes in H3K9me3 at the periphery could be responsible for changes of nuclear peripheral heterochromatin contribution to nuclear mechanics. To determine if H3K9me3 is also lost at the periphery, we measured 1 µm rim of the nuclear periphery for H3K9me3 signal across conditions. To our surprise, Chaetocin treatment did not alter H3K9me3 levels at the nuclear periphery. Though we would like to note that nuclei were highly variable with some showing loss, while others showed increased peripheral staining. Loss of H3K9me2 resulted in a significant increase in H3K9me3 periphery intensity relative to wild type, while the loss of H3K27me3 did not measure a significant change (Figure 5(f)). H3K9me3 periphery remains unchanged upon Chaetocin treatment while chromocenter compaction decreases drastically suggesting it is the mechanism for observed changes in nuclear mechanics, morphology, and integrity.

## Discussion

Heterochromatin compaction controls nuclear mechanics, though the different roles of core subtypes and histone modifications have been elusive. Using established histone methylation inhibitors, we separately decreased constitutive heterochromatin histone modification H3K9 di- and tri-methylation. We find that H3K9me3 provides essential chromatin compaction to chromocenters that dictates nuclear mechanics and thus morphology and integrity. H3K9me2 has the opposite effect counteracting over-compaction of both chromocenters and the nuclear periphery. We reveal that constitutive heterochromatin structures are not altered by changes in facultative heterochromatin, while facultative heterochromatin has a similarly important role in nuclear mechanics, morphology, and integrity. Overall, we provide a detailed separation of core histone modifications roles in chromatin compaction and nuclear mechanobiology.

### H3K9me2 is an anti-compaction mechanism

Our data reveals that H3K9 dimethylation is an anti-compaction mechanism. Loss of H3K9me2 results in an increase in both chromocenter and peripheral H3K9me3 levels and compaction, increased chromatin-based nuclear mechanics, and better maintenance of nuclear shape and integrity (Figures 2, 4, 5). Previous studies have shown that H3K9me2 antagonizes H3K9me3 spreading [59]. Thus, H3K9me2 anti-H3K9me3 activity can modulate constitutive heterochromatin compaction and its role in nuclear rigidity. This H3K9me3 anti-spreading also likely includes H3K9me3 recruitment of chromatin linker HP1α, a known mechanical contributor. Furthermore, while studies have reported that HP1α binds H3K9me2, loss of H3K9me2 shows an increase in HP1α enrichment. While it might have been hypothesized that loss H3K9me2 would lead to a decrease in HP1α levels, our data show the opposite upon BIX treatment. In summary, we show novel data that H3K9me2 is a chromatin decompaction and softening component that when lost causes increased nuclear mechanics and better nuclear shape and integrity maintenance through increased chromocenter compaction and rigidity.

### H3K9me3 chromocenter compaction controls constitutive heterochromatin chromatin-based nuclear mechanics, morphology, and integrity

H3K9me3 provides essential compaction and rigidity to constitutive heterochromatin in the nucleus. Specifically, SUV39H1-based H3K9me3 is responsible for maintaining chromocenter compaction to control chromatin-based nuclear mechanics. On the flip side, the loss of H3K9me2 via BIX increased H3K9me3 chromocenter compaction and chromatin-based nuclear mechanics. Thus, our data show the effect of modulating H3K9me3 in both directions, a powerful set of data clarifying the mechanism underlying constitutive heterochromatin compaction and rigidity. Finally, the loss of H3K27me3 did not impact chromocenter compaction further, indicating that chromocenter compaction is controlled by constitutive heterochromatin histone modification via H3K9 methylation.

H3K9me3 controls of chromocenter compaction is independent of HP1α. Loss of HP1α chromocenter enrichment occurs in both loss of H3K9me3 via Chaetocin and H3K27me3 via DZNep. In DZNep-treated cells chromocenters remain compact showing that the loss of HP1α and/or H3K27me3 does not alter the compaction (Figure 5(d,e)). Our data supports that chromocenter compaction is reliant on core H3K9me3 because that is the only treatment that measured decreased compaction (Figure 5(d) and Supplemental Figure S2e). Oppositely, peripheral H3K9me3 did not significantly change upon loss of H3K9me3 via Chaetocin (Figure 5(f)). Thus, our data provide a clear mechanism of H3K9me3 in maintaining chromocenter compaction to provide the nuclear mechanical rigidity of constitutive heterochromatin.

H3K9me3 and HP1α contribute to separate chromatin functions within the mechanical chromatin polymer gel. We previously showed that H3K9 methylation has a separable mechanical contribution from HP1α which acts as a chromatin cross linker [30,31]. In our physics-based simulation models of nuclear mechanics histone modification state dictates compaction, which is a separate parameter from chromatin–chromatin crosslinks. Our previous work revealed two types of mechanical chromatin cross linkers HP1α and chromosome interactions measured by Hi-C [30,31,33]. The requirement of H3K9me3 to maintain chromatin compaction confirms that histone modifications control compaction, while HP1α is dispensable. Thus, our data support that H3K9me3 chromocenter compaction dictates both nuclear mechanics and HP1α chromocenter enrichment. This agrees with previous work showing chromocenter compaction is independent of HP1α [58]. While HP1α enrichment is lost upon inhibition of either H3K9me3 or H3K27me3, the global level remains the same. This suggests that HP1α is still present and actively forming chromatin cross links to aid nuclear mechanics, though its displacements from chromocenter chromatin interaction hubs may make HP1α less efficient. Thus, H3K9me2 antagonizes and H3K9me3 contributes to chromocenter compaction, which is the primary driver of nuclear mechanics, while HP1α’s separable mechanical contribution likely provides a minimal secondary downstream effect.

Weaking of the nuclear spring constant due to the loss of constitutive heterochromatin H3K9me3 drives nuclear blebbing and rupture. Nuclear blebbing is largely believed to be a force balance between actin and/or microtubule deformation forces vs. nuclear mechanical resistance. However, this idea has seen recent changes largely sparked by the fact that nuclear blebbing has been shown to be reliant on transcriptional activity. Modulation of transcriptional activity does not alter nuclear mechanics [22] but alters micron-scale coherent motion [60–62]. Modeling suggests chromatin motion and cross-links can control nuclear shape fluctuations [63] with models showing periphery chromatin alone can control gross nuclear shape and fluctuations [64]. While we measure no change in global peripheral H3K9me3, local defects especially at sites of high curvature may aid nuclear blebbing and rupture. Decoupling the relative roles of bulk mechanical changes and local changes to nuclear blebbing will require ongoing future studies. Overall, we find an interesting interplay between H3K9me2 antagonism of H3K9me3 that largely manifests as changes in chromatin-based nuclear mechanics, blebbing, and rupture.

### H3K9me3 role in lamin-based strain stiffening

Loss of H3K9me3 uniquely causes loss of nuclear mechanics at both the chromatin-based short and lamin-based long extension regimes. The current view of nuclear mechanics has chromatin as the base layer of stiffness, while lamins become engaged at longer extension to create strain stiffening [50,56,57,65–67]. These data report a novel behavior that a chromatin perturbation results in changes in the lamin-based strain stiffening regime. Loss of H3K27me3 via DZNep did not result in this behavior (Figure 4). Furthermore, previous publications modifying H3K9ac via HDACi and other histone based-modifications also reported no significant change in the lamin-based strain stiffening regime [14,21,22]. Loss of HP1α decreases nuclear mechanics leading to abnormal nuclear shape and rupture but does not alter the strain stiffening lamin-based regime [30]. Finally, the loss of lamin A/C also does not occur upon loss of H3K9me3 in Chaetocin treated cells (Supplemental Figure S1b). Thus, H3K9me3 has a unique role in contributing to the lamin-based strain stiffening.

One hypothesis is that H3K9me3 disrupts chromatin-lamin linkages. Loss of a chromatin-lamin linker has been shown in simulations to have the capability to decrease both the short chromatin-based regime as well as the long extension strain stiffening by lamins [30]. Oppositely, chromatin compaction and chromatin–chromatin interactions do not impact lamin-based strain stiffening by simulation [30,65] which is supported by experimental data listed early, including HDACi, DZNep, and HP1α degradation. While we measure no change in the levels of peripheral H3K9me3 upon its inhibition with Chaetocin (Figure 5(f)), this measurement is not a direct measure of chromatin-lamin linkages. Future work directly measuring chromatin to lamin linkages via FISH [68] or DAM-ID [69] would help clarify the mechanism. H3K9me3 is known to have interactions, with possible chromatin lamin linking proteins including PRR14 [70,71]. It is also possible that while lamin A/C levels measured no change, levels of lamin incorporation might be altered which could impact lamin-based nuclear mechanics. The mechanism underlying the role of H3K9me3 in lamin-based strain stiffening will require further experiments to test this hypothesis directly.

An alternative hypothesis is that lamin-based strain stiffening is partially a function of chromatin stiffness. The data partially support this hypothesis because throughout the four measured conditions the long:short regime ratio remains relatively constant at a two-fold increase (Figure 4(g)). However, no other chromatin perturbation to date has altered lamin-based strain stiffening, suggesting there is a key difference between H3K9me3 and other chromatin modifications and/or proteins. Again, future work will be needed to address these hypotheses.

### Differences between the roles of constitutive and facultative heterochromatin

When comparing the roles of constitutive and facultative heterochromatin, both are similarly important for maintaining chromatin-based nuclear mechanics, morphology, and integrity. Differences arise in structure. Specifically, our data show that constitutive heterochromatin H3K9me3 supports chromocenter compaction, while facultative is dispensable. This agrees with their differential localization via immunofluorescence and their well reported mutually exclusive behaviors in the genome [32,72]. Many newer studies suggest that H3K27me3 can compensate for the loss of H3K9me3 [73,74]. However, this does not appear to be the case for either nuclear mechanics or chromocenter compaction upon the loss of H3K9me3 via Chaetocin (Figures 4 and 5). One clear difference could be that upon loss of facultative heterochromatin transcription levels would increase, which we recently reported drives nuclear blebbing and rupture [22]. While this might explain the slightly higher levels of nuclear blebbing and rupture upon loss of H3K27me3, transcription changes do not alter nuclear mechanics. Facultative heterochromatin H3K27me3 has been shown to be important for super silencers driven by chromatin–chromatin looping interactions [75]. Future studies will be required to determine how facultative heterochromatin marker H3K27me3 maintains chromatin structure that is responsible for its role in chromatin-based nuclear mechanics, morphology, and integrity.

Another major difference is that constitutive heterochromatin is essential for proper mitosis, while facultative is not. Our data show that 60% of mitosis show abnormality upon loss of H3K9me3 (Figure 3) has been well reported for almost two decades [51–53]. Loss of H3K9me2 or H3K27me3 did not significantly alter mitotic outcome. We report that the loss of constitutive heterochromatin H3K9me3 mitotic failures results in abnormally shaped nuclei. This mitotic effect is separate from the interphase-based H3K9me3 loss resulting in nuclear weakening, blebbing, and rupture. Thus, loss of constitutive heterochromatin can cause dysfunction in non-dividing cells via its interphase-based nuclear mechanics, morphology, and integrity effects while also causing dysfunction in dividing cells via its mitosis-based roles. Overall, the loss of constitutive heterochromatin H3K9me3 marker shows both clear similarities and differences with the loss of facultative heterochromatin H3K27me3. Specifically, constitutive heterochromatin H3K9 methylation controls chromocenter compaction to influence nuclear mechanics, shape, and integrity.

## Materials and methods

### Cell culture

Mouse Embryonic Fibroblasts (MEF) cells and Human HT1080 cells (ATCC) were cultured in DMEM (Corning) complete with 10% fetal bovine serum (FBS; HyClone) and 1% penicillin/streptomycin (Corning). The cells were incubated at 37°C and 5% CO2 and passaged every 2–3 days.

### Drug treatment

Cells were first plated in DMEM complete to 50% confluence. Then, cells were incubated overnight with 20 nM of Chaetocin (13156, CaymanChem), 1 µM of BIX01294 (72042, Stemcell Technologies), or 2.5 µM of DZNep (50-606 -90,001, Fisher) for 16–24 h. To modify actin contraction, we used 10 µM Y27632 (129830-38-2, Tocris) for 16–24 h and 10 nM of Rho Activator II CN03 (Cytoskeleton) for 3 h.

### Immunofluorescence

MEF cells were grown in 8-well glass chambers (Cellvis) and treated as above. Cells were fixed with 4% paraformaldehyde (Electron Microscopy Sciences) in phosphate-buffered saline (PBS; Corning) for 15 min. Following fixation, cells were washed three times with PBS for 5 min intervals. Cells were permeabilized with 0.1% Triton X-100 (Promega) for 15 min and washed with 0.06% Tween-20 (PBS-T) for 5 min followed by three PBS washes for 5 min each. Cells were then blocked with 2% bovine serum albumin (BSA; Fisher Scientific) in PBS and allowed to sit at room temperature for 1 h.

Primary antibodies were diluted in the blocking solution at the following concentrations: Lamin A/C 1:1,000 (4777, Cell Signaling Technologies), H3K9me3 1:1,000 (8898, Abcam), H3K9me2 1:500 (4658, Cell Signaling Technologies), H3K27me3 1:600 (9733, Cell Signaling Technologies), Lamin A/C 1:200 (4C11, Cell Signaling Technology), pMLC2 1:100 (3672, Cell Signaling Technologies), and HP1α 1:250 (109028, Abcam). After placing in the primary antibodies, the 8-well glass chamber was wrapped in foil and allowed to sit at room temperature for 2 h. Primary antibodies were aspirated and washed three times with PBS for 5 min intervals.

Secondary antibodies were added to the dish and allowed to sit for 1 h at room temperature. Secondary antibodies used were Alexa Fluor 647 Anti-Rabbit or Anti-Mouse IgG 1:1,000 (4414, Cell Signaling Technologies) and Alexa Fluor 555 Anti-Rabbit 1:1,000 (4413, Cell Signaling Technologies). Secondary antibodies were then aspirated, and the dish was washed with PBS three times for 5 min intervals. The cells were stained with Hoechst 1:10,000 (33342, Invitrogen) in PBS for 15 min and washed with PBS three times for 5-min intervals.

### Immunofluorescence imaging and analysis

Following staining of fixed cells, imaging was conducted using Nikon Eclipse Ti2 microscope with 40× Plan Abo (N.A 0.75, W.D. 0.66, MRH00401). Image stack parameters for immunofluorescence imaging were 0.5 µm z-steps over 4.5 µm (nine steps) over six fields of view per condition.

Within the NIS-Elements AR Analysis software, z-stacks were compiled into a maximum projection and average background fluorescence was subtracted from each field of view using a 30 × 30 pixel square area containing no cells. Individual nuclei were selected using auto-selected ROIs. The average intensity of the associated channel was acquired for each nuclei selected. Average intensities were exported to Microsoft Excel to determine the relative levels of heterochromatin, euchromatin, HP1α, and lamina between conditions. Statistical significance between average Cy5 intensity between conditions was determined using a one-way ANOVA multiple comparisons.

As previously outlined [7] the pMLC2 immunofluorescence analysis was conducted via Z-stacks compiled into a maximum projection. To capture the average intensity of pMLC2, a 30 × 30-pixel ROI was drawn around each nucleus. The background intensity was measured via an ROI drawn in an area absent of nuclei. The average Cy5 intensity of each nuclei’s ROI as well as the background ROI was exported into Excel. In Excel, the background intensity was subtracted from each ROI to determine the average levels of intensity of pMLC2 per nuclei. The average intensities of 20 nuclei per condition were exported into PRISM where a normality test was run on each condition. The data set did not pass the normality test resulting in statistical significance being determined by the Student's t-test.

### Time lapse imaging and analysis

Images were captured with Nikon Elements software on a Nikon Instruments Ti2-E microscope with 40× Plan Abo Lambda objective (N.A 0.75, W.D. 0.66, MRH00401). Instruments include the Ocra Fusion Gen III camera, Lumencor Aura III light engine, TMC CleanBench air table.

Live cell time lapse imaging was conducted using Nikon Perfect Focus System and Okolab heat, humidity, and CO2 stage top incubator (H301). Cells were treated with histone modification drugs as described above 8 h prior to imaging. Time lapse imaging parameters for MEF cells were: 3-min intervals for 16 h with six fields of view per condition, 3% FITC Wide Field power, and 30 ms exposure time. Parameters for HT1080 cells were: 3-min intervals for 16 h with six fields of view per condition, 5% TRITC Wide Field power, 3% FITC Wide Field power, and 30 ms exposure time. Within the NIS-Elements AR Analysis software, each field of view was observed to record total number of nuclei, number of blebs, numbers of ruptures, and mitotic events. For rupture data, recorded events included: number of bleb-based or non-bleb-based ruptures, rupture frequency, and cell cycle stage of rupture (pre- or post-mitosis). Data was compiled and averaged in Microsoft Excel to compute percentages of bleb and rupture conditions for each field of view. To gain circularity measurements, ROIs were drawn around daughter nuclei post mitosis. The circularity measurements were pulled at 5 to 10 frames or 15–30 min after nuclei balled up to undergo mitosis. Statistical significance for all measurements was determined using a Student's t-test.

### Micromanipulation force measurement of an isolated nucleus

As previously described [29,55], MEF Vimentin null (MEF V-/-) were grown in a micromanipulation well and treated as described above. Nuclei were isolated from living cells using a spray micropipette containing Triton X-100 (0.05%) in PBS. A pull micropipette was used to grab the nucleus, while the opposite end of the nucleus was grabbed by a precalibrated force micropipette and suspended in preparation for force-extension measurements. Micropipettes are fashioned using a Flaming/Brown micropipette puller *p*-97 (Sutter Instruments) using pipettes from World Precision Instruments TW100–6 for pull and spray pipettes and TW100F–6 for force pipettes. Pull and spray pipettes were fabricated with a pull program of Heat 564, Pull 110, Velocity 110, Time 100, and Pressure 500. Force pipettes used a pulling program of Heat 561, Pull 220, Velocity 200, Time 20, and Pressure 500. Pulling completes with the separation of the capillary into two pipettes, which are cut with a heated filament to an ideal width of 3–6 µm. The pull pipette was moved 50 nm/s to extend the nucleus 3 or 6 µm. Both the pull and force pipettes positions were tracked with our established LabView written program. The pipette positions at each time interval are output to excel to extract analysis. Nucleus extension was measured by tracking the change in distance (µm) between the pull and force micropipettes. Force (nN) was measured by using Hooke’s law F = kx, where x is the deflection distance (µm) of the force pipettes from its initial position multiplied by k the precalibrated bending modulus (nN/µm). This force pipette was precalibrated to set range of 1.2–2 nN/µm bending modulus. In excel, the force vs extension is plotted. The slope of the force versus extension plot provides the spring constant (nN/µm) for the short chromatin-dominated regime (<3 µm) and long-extension lamin A-dominated strain-stiffening regime (>3 µm). Each nucleus is force-extension measured three times and averaged. The long-regime spring constant minus the short-regime spring constant provides the measure of lamin A-based strain stiffening.

### Chromocenter and periphery chromatin measurement

As previously described in [7], spinning disk confocal images were acquired with Nikon Elements software on a Nikon Instruments Ti2-E microscope with Crest V3 Spinning Disk Confocal, Orca Fusion Gen III camera, Lumencor Celesta light engine, TMC CleanBench air table, with Plan Apochromat Lambda 100× Oil Immersion Objective Lens (N.A. 1.45, W.D. 0.13 mm, F.O.V. 25 mm, MRD71970). Z-stacks were compiled into a maximum projection and average background fluorescence was subtracted for each field of view via an ROI pixel area with no cells. For chromocenter analysis in H3K9me3 and HP1α immunofluorescence images, NIS-Elements were used to auto select ROIs of all the chromocenters in each nucleus to capture average intensity of Cy5 fluorescence, area of ROI, and size of nuclei. The measurements from the NIS-Elements software were then exported to Excel.

To measure the intensity of the periphery of the nuclei, the previously acquired H3K9me3 IF images were analyzed. For each of the nine fields of view, Z-stacks were compiled into a maximum projection and the average background fluorescence was subtracted via an ROI pixel area with no cells. Using NIS-Elements, individual nuclei were auto selected as ROIs in binary. The ROIs were then eroded by 15 pixels, or 1 µm, and converted into separate ROIs. The measurements of the two ROIs per nuclei were exported to Excel. To provide the peripheral intensity measurement for each nucleus, the average Cy5 intensity of the eroded ROI was subtracted from the average Cy5 intensity of the whole nucleus.

### Statistics

Each data set was measured for normality via the Shapiro–Wilk test. Data sets that were normally distributed with homogenous variance (tested via the Welch’s ANOVA test) were then run for statistical significance via a one-way ANOVA multiple comparisons test (Brown-Forysthe and Welch) in PRISM. Those data sets that did not meet the normality assumption were then analyzed for statistical significance via the non-parametric Mann–Whitney U test. Furthermore, data sets that could not be reliably tested for normality due to limited numbers of replicates (data sets with only 3 replicates per condition) were run for statistical significance via a Student’s t-test. A low number of replicates is non-advisable to test for normality. Each figure legend specifies which statistical test was used on the data. In short, one-way ANOVA multiple comparison tests were run on Figures 1, 4(g), 5(e,f), and Supplemental Figures S1b,c, S2e,f. Mann–Whitney U tests were run on Figures 4(d-f), 5(b-d), and Supplemental Figures S1a, S2g, and S3a-b. Two-tailed unpaired Student’s t-tests were run on Figures 2 and 3 which consisted of data points from biological replicates [76].

## Supplementary Material

Supplemental Material

## Data Availability

Raw data is available via FigShare Doi: https://doi.org/10.6084/m9.figshare.27241950 All microscope data sets are available upon request due to the size of these files.
