# Supplementary material for: Constitutive heterochromatin controls nuclear mechanics, morphology, and integrity through H3K9me3 mediated chromocenter compaction
Source: Nucleus. 2025 Apr 9;16(1):2486816. doi: 10.1080/19491034.2025.2486816 (PMC11988277; doi:10.1080/19491034.2025.2486816)
Supplement: Supplemental Material [file KNCL_A_2486816_SM1003.zip › Supplemental Figure Caption.docx]

**Supplemental Figure 1. Phospho-MLC2, lamin A/C, and HP1α levels remain unchanged by inhibitors altering histone modifications.** (A) Example immunofluorescence images and graph of relative levels of (A) pMCL2 (white) and DNA via Hoechst, (B) lamin A/C, and (C) HP1α Each condition consists of (A) 20 nuclei and (B, C) 6 replicates with n > 20 cells with a total of n > 150 cells for each condition. Data was analyzed via (A) Mann-Whitney U test or (B, C) one-way ANOVA multiple comparison with p values reported as * < 0.05. Error bars represent standard error. Scale bar = 10 µm.

**Alt text:** Images of immunofluorescence examples for cellular pMLC2, nuclear lamin A/C, and nuclear HP1α. Scatter plot or bar graph of average immunofluorescence intensity relative to wild type show no changes for histone modification inhibitors BIX, Chaetocin, and DZNep.

**Supplemental Figure 2. H3K9me3 chromocenter heatmaps, DNA compaction, and nucleus characteristics.**  Chromocenter H3K9me3 average intensity and size graphed as a heatmap where min and max determine 10 major color differences on a spectrum of 100 color variations corresponding to density as shown by the increasing density bar. This data is graphed for our major conditions (A) wild type, (B) BIX01294, (C) Chaetocin, and (D) DZNep. Respectively, each condition consists of n chromocenters where WT n = 702, BIX n = 470, Chaetocin n = 491, and DZNep n = 635. Graphs of (E) DNA chromocenter compaction, (F) nuclear size, and (G) chromocenters per nucleus from the H3K9me3 chromocenters used in Figure 5, in which 30 nuclei were measured for each condition. A one-way ANOVA multiple comparison was used for panels E and F and Mann-Whitney U test for panel G between wild type and each condition p values reported as * < 0.05, ** < 0.01, *** < 0.001, or no asterisk denotes no significance, p > 0.05. This raw data is available in **Supplemental Data 1**.

**Alt text:** Individual heatmaps showing chromocenter H3K9me3 average intensity and size for each major condition. Scatter plot graph of average DNA chromocenter compaction showing an increase with BIX01294 treatment, decrease with Chaetocin, and decrease with DZNep. Scatter plot graph of average nuclear size showing only a decrease in BIX01294 and increase in DZNep. Scatter plot graph of average of the number of chromocenters per nucleus showing a decrease with both BIX01294 and Chaetocin treatment.

**Supplemental Figure 3. Nuclear blebbing and chromocenter compaction are restored upon dual knockdown of H3K9me2 and H3K27me3.**  (A) Nuclear blebbing percentages for wild type (WT, black), BIX01294 (BIX, blue),and DZNep (purple), and BIX + DZNep (perwinkle) for 9 technical replicates average n > 30 each replicate. Examples images of normal and blebbed nuclei shown the right via DNA stained with Hoechst. (B) Graphs of relative chromocenter intensity per area for wild type and BIX+DZNep for both H3K9me3 and HP1α, in which 30 nuclei were measured for each condition. Example images of HP1α chromocenters shown the right. Mann-Whitney U test between all treatments with p values reported as * < 0.05, ** < 0.01, *** < 0.001, or no asterisk denotes no significance, p > 0.05. Error bars represent standard error. Scale bar = 10 µm.

**Alt text:** Images of normal, ellipse nuclei and blebbed nuclei. Bar graph of average percent nuclear blebbing showing decreased blebbing with BIX01294, increased blebbing with DZNep, and wild type levels with BIX + DZNep dual treatment. Example images of wild type and BIX + DZNep nuclei immunofluorescence of HP1α. Scatter plot of average and individual relative chromocenter intensity per area of WT and BIX + DZNep showing no significance difference in either H3K9me3 or HP1α.
